# Virulence gene profiles of extraintestinal pathogenic Escherichia coli (ExPEC) from Zambia: a secondary in silico study

**DOI:** 10.1099/acmi.0.001165.v3

**Published:** 2026-09-22

**Authors:** Frank Chilombolwa Nyondo

**Affiliations:** 1 Department of Biomedical Sciences, Ridgeway Campus, University of Zambia, School of Health Sciences, Lusaka, Zambia

**Keywords:** *Escherichia coli*, extraintestinal pathogenic *E. coli*, prophages, replicons, Sequence Type 131, virulence genes

## Abstract

This secondary *in silico* study analysed 36 *Escherichia coli* draft genome assemblies from Zambia to characterize the virulence gene profiles of extraintestinal pathogenic *E. coli* (ExPEC) and to relate them to mobilome proxies, namely plasmid replicons and prophage content. All assemblies met plausibility thresholds for *E. coli* (1–17 contigs; 4.85–5.77 Mb; G+C 50.47–50.96 mol%), and species identity was confirmed by average nucleotide identity to the *E. coli* type strain (96.6–98.9%). Using the Achtman seven-locus MLST scheme applied to the reads, 35 of 36 genomes were assigned a sequence type (ST), and 1 remained untypeable at a single locus. The most frequent type was ST69 (9 out of 36) rather than the globally dominant ST131 (6 out of 36), followed by ST405 (5 out of 36) and ST617 (5 out of 36). Phylogroup D was most frequent (12 out of 36), followed by the commensal-associated phylogroup A (8 out of 36) and the pandemic phylogroup B2 (6 out of 36). Classical intestinal-pathotype hallmarks (*stx1*, *stx2*, *eae*, *bfpA* and *ipaH*) were not detected. In contrast, ExPEC-associated markers were common, including *iutA* (27 out of 36), *iss2* (26 out of 36), *fyuA* (24 out of 36), *kpsM* (23 out of 36) and *ompT* (18 out of 36), with a high within-genome burden (median ~193 loci; range 173–251). Gene-based rules classified 19 of 36 genomes as ExPEC and 2 as enteroaggregative *E. coli*, and source-informed analysis identified uropathogenic, neonatal meningitis-associated and sepsis-associated subpathotypes. IncF-family replicons predominated, *sitABCD* occurred in both chromosomal and IncF-plasmid contexts, and *iss2* was recurrently prophage-associated. These findings indicate that extraintestinal virulence in this population is distributed and modular rather than confined to ST131. This virulence is carried across diverse lineages and phylogroups, including classically commensal backgrounds, and is mobilized by plasmids and prophages. Ultimately, these data provide a baseline for the genomic surveillance of virulent *E. coli* in Zambia.

**Impact Statement**

Extraintestinal pathogenic *Escherichia coli* can leave the gut and cause urinary tract infections, bloodstream infections and neonatal meningitis. In Africa, most genomic studies on *E. coli* have focused on antimicrobial resistance (AMR), especially on widely spread lineages such as ST131, that often carry extended-spectrum *β*-lactamase genes. This study uses publicly available Zambian whole-genome data to shift the focus to virulence, the genetic features that help *E. coli* acquire scarce nutrients such as iron, survive in blood or urine and evade immune defences. It also examines the mobilome – the plasmids and prophages that can transfer these traits between strains. Across diverse sequence types, phylogroups and sample sources, genes for iron acquisition, serum survival and capsule formation were common, while classic enteric toxin markers were absent. Notably, this virulence was not confined to ST131 but was most frequent in ST69 and extended even to lineages usually regarded as harmless gut commensals. By connecting lineage structure with virulence traits and mobile genetic elements, this work establishes the first Zambian baseline for virulence-focused genomic surveillance and shows why focusing solely on AMR may overlook traits that affect infection risk and spread.

## Data Summary

Whole-genome sequencing data analysed in this study were retrieved from public repositories (NCBI BioProject: https://www.ncbi.nlm.nih.gov/bioproject/PRJDB10450) and were initially generated and deposited by Shawa *et al*. [1]. The specimen metadata and accessions, sequence typing, virulence-gene screen, pathotype classification and plasmid replicon and prophage analyses are provided as Tables S1–S8, available in the online Supplementary Material, in a single combined workbook deposited alongside this manuscript. No new sequencing data were generated. All intermediate outputs derived from these assemblies (MLST calls, virulence screening tables, plasmid replicon screening tables, prophage predictions and derived presence/absence matrices) are provided as supplementary files (https://doi.org/10.6084/m9.figshare.30964066) and/or deposited alongside this manuscript. All software used (including versions) and the reproducible command history/scripts used to run analyses and generate figures are provided in the supplementary reproducibility materials accompanying this submission (https://doi.org/10.6084/m9.figshare.30964066). The same materials are maintained in a public code repository at https://github.com/chilonyondo-cyber/Zam_Virolome_Ecoligenomics, and the figshare deposit is the citable archived version of that repository.

## Introduction

*Escherichia coli is* a Gram-negative enteric bacterium within the family *Enterobacteriaceae* that demonstrates extensive diversity, ranging from harmless gut commensals to strains capable of causing severe disease [2]. Beyond diarrhoeal illness, *E. coli* is a significant cause of extraintestinal infections, including urinary tract infections (UTIs), bloodstream infections (BSIs) and neonatal meningitis. Notably, the clinical outcomes of these infections are increasingly complicated by antimicrobial resistance (AMR) [3]. In the context of extraintestinal disease, extraintestinal pathogenic *E. coli* (ExPEC) refers to strains with genomic or experimental features that facilitate survival and pathogenesis outside the gut [4]. Importantly, ExPEC is not a single clone or classical pathotype; rather, it is a functional category comprising multiple lineages that have acquired various combinations of virulence determinants. These genes enhance colonization, immune evasion, nutrient acquisition, tissue damage or dissemination in extraintestinal sites [5, 6].

One of the major challenges in understanding *E. coli* genomics is that important clinical phenotypes arise from different combinations of genes. To address this complexity, two broad frameworks are commonly applied. The first distinguishes intestinal pathogenic *E. coli* (IPEC), which includes diarrhoeagenic pathotypes such as ETEC, EPEC, EAEC, STEC and EIEC, defined by specific regulators, toxins and adhesins. The second framework, however, is more relevant for ExPEC: ExPEC-associated syndromes, including uropathogenic *E. coli* (UPEC)*,* neonatal meningitis-associated *E. coli* (NMEC) and sepsis-associated ExPEC, are typically identified by marker sets enriched among isolates from these clinical presentations [7, 8]. The virulome, defined as the set of virulence genes in a genome, is examined in ExPEC to characterize how these genes vary and co-occur across isolates, lineages and sources [6, 8]. While identifying virulence genes in genomic data establishes the biological plausibility for pathogenesis, it does not directly equate to demonstrated *in vivo* virulence. Actual ExPEC disease outcomes are governed by a complex interplay of downstream factors, including gene expression, regulation, host susceptibility and the specific route of infection [3, 9].

Multiple studies consistently identify several virulence functions within ExPEC. For example, iron acquisition systems, including those associated with aerobactin (*iucABCD* and *iutA* genes) and yersiniabactin (*irp*, *ybt and fyu* genes), facilitate growth in iron-limited environments such as urine and blood [10, 11]. In addition, serum survival and complement evasion mechanisms promote persistence during bacteraemia, while capsule or surface structures enhance immune evasion and dissemination [6, 12]. These modules are subject to horizontal transfer, meaning ExPEC-associated traits are distributed across diverse phylogenetic backgrounds [3, 5]. Thus, both lineage context, such as MLST sequence types (STs), and gene-content context are informative. MLST provides a stable framework for population structure, while virulence profiles reveal functional potential within and between lineages [5, 8, 13].

ST131 is a prominent *E. coli* lineage in the context of AMR. It is globally distributed and closely associated with multidrug resistance and the dissemination of extended-spectrum *β*-lactamase (ESBL) genes, particularly CTX-M enzymes, in both hospital and community settings [5, 7, 14, 15]. As a result, ST131 has significant public health implications due to its efficient spread, persistence, frequent carriage of ESBL resistance genes and possession of virulence traits often described as ‘ExPEC-like’ [4, 14, 15]. A critical question, especially in regions with limited genomic surveillance, is whether ExPEC-related virulence is primarily concentrated in a few high-impact clones such as ST131 or is more broadly distributed among local *E. coli* populations with diverse STs and combinations of extraintestinal fitness traits [3, 5, 6].

Globally and in Africa, most *E. coli* genomic research has prioritized tracking AMR, particularly the spread of ESBLs and the monitoring of well-characterized lineages that disseminate these resistance genes [7, 14, 16]. This emphasis is justified by the substantial clinical impact of ESBL-related treatment failures and the widespread occurrence of ESBL-producing *E. coli* in communities, hospitals, animals and the environment [16, 17]. In Zambia, studies have documented ESBL and AMR in *E. coli* from diverse sources, including patients, animals and food samples [1, 18–20]. However, research on the virulence of Zambian *E. coli*, particularly studies integrating virulence profiles with data on mobile genetic elements, remains limited. This gap hinders the ability of current surveillance to identify lineages that also possess gene combinations conferring extraintestinal survival [3, 5, 16].

This imbalance has a second dimension. Across sub-Saharan Africa, genomic research on *E. coli* has concentrated heavily on the diarrhoeagenic pathotypes, reflecting the large paediatric diarrhoeal disease burden, while the virulence gene content of extraintestinal isolates has been described far less often [5, 21]. In Zambia, the local focus has similarly fallen on diarrhoeagenic and enterotoxigenic isolates from children [22–24]. The global literature compounds this imbalance, since genomic effort has concentrated on the pandemic clone ST131 while other extraintestinal lineages remain comparatively understudied [25]. Characterizing the virulome of extraintestinal isolates, therefore, addresses a recognized gap and provides part of the rationale for the present analysis.

Virulence and AMR should be considered together because both are frequently influenced by the mobilome, which consists of mobile genetic elements that transfer within and between genomes [26]. In *E. coli*, this primarily involves plasmids, which can carry both resistance and virulence modules, and prophages, which are integrated bacteriophage genomes capable of introducing accessory genes or altering bacterial biology through regulatory effects [6, 17, 27]. As a result, the mobilome provides a mechanistic basis for the dissemination of ExPEC-associated traits, such as iron acquisition and serum survival, as well as AMR determinants across different STs, resulting in mosaics of fitness traits not confined to a single clone [3, 6, 17]. In epidemiological studies, plasmid replicon types are often used as proxies for plasmid backbone families, while prophage analyses typically assess burden and putative gene content rather than reconstructing transmission phylogenies, particularly when using draft genome assemblies [28].

In this context, this study utilizes publicly available whole-genome sequence assemblies originally generated by Shawa *et al*. [1] to investigate the virulence architecture of Zambian *E. coli*. This approach complements the AMR-focused perspective that has predominated in regional *E. coli* genomics research [1, 14, 19, 20]. Specifically, this secondary analysis characterizes the distribution of ExPEC-associated virulence determinants across various STs and specimen sources. Furthermore, it evaluates how genomic features, such as plasmid replicon backgrounds and prophage signals, influence these observed gene-content patterns. By linking virulence profiles to lineage structure and mobilome context, the study aims to establish a baseline for the distribution of extraintestinal fitness traits in Zambian *E. coli* populations and to encourage future research that integrates comprehensive clinical metadata and prospective sampling with genomic surveillance.

## Methods

### Study design and data sources

This study presents a retrospective, *in silico* secondary analysis of a previously published collection of cefotaxime-resistant Enterobacteriaceae from the University Teaching Hospital (UTH), Lusaka, Zambia [1]. The parent study included 46 non-repetitive clinical isolates collected between June and October 2018 from routine specimens such as stool, urine, sputum, pus, cerebrospinal fluid (CSF), blood and high vaginal swabs (Table S1). Whole-genome sequencing in the parent study utilized Illumina MiSeq short reads and Oxford Nanopore MinION long reads, followed by hybrid assembly and polishing [1].

For this secondary analysis, assembled genome FASTA files corresponding to the *E. coli* subset (36 genomes; isolate IDs Zam_UTH_01–Zam_UTH_51, as available) were retrieved from the public repositories referenced in the parent study and are summarized in Table S2.

### Assembly-level quality control

Assembly quality control (QC) was conducted directly on the downloaded FASTA assemblies using SeqKit v2.12.0 [29]. Summary metrics, including contig count, total assembly length and G+C content, were calculated using seqkit stats -a and parsed with shell and awk utilities [29].

Assemblies were evaluated against established *E. coli* plausibility thresholds: genome size, 4.5–6.2 Mb; G+C content, 49–52 mol%; and contig count, ≤200 [30]. Among the 36 assemblies analysed, observed ranges were contigs, 1–17; genome size, 4,848,171–5,768,673 bp; and G+C content, 50.47–50.96 mol% (File S2).

### MLST

STs were determined directly from the sequencing reads using ARIBA v2.14.7 [31], with assembly-based calling using mlst v2.33.1 retained as a cross-check [32] against the Achtman seven-locus *E. coli* scheme (ecoli_achtman_4) [13]. To resolve potentially ambiguous or incomplete locus calls within fragmented assemblies, read-based allele calling was conducted using ARIBA to capture sequences spanning contig boundaries. These calls were independently verified via assembly-based typing using mlst (blastn v2.17.0+). In cases of discordant or novel allele assignments, final designations were adjudicated through direct inspection [33]. When allele calls were ambiguous, such as when multiple close allele matches were indicated by a question mark, isolates were flagged as ambiguous for downstream tabulation rather than being assigned a definitive ST.

### Phylogrouping, serotyping and species confirmation

Phylogroups were assigned with EzClermont v1.0.0 [34, 35], an *in silico* implementation of the Clermont quadruplex scheme, applied to each genome assembly. Genomes for which the control or typing loci could not be resolved by the scheme were recorded as phylogroup-unassigned and were retained in all downstream analyses. Serotypes, comprising O and H antigens, were predicted with ECTyper [36] v2.0.0 running database v1.0. Species identity was confirmed by average nucleotide identity calculated with FastANI [37] v1.34 against the *E. coli* type strain ATCC 11775 (assembly accession GCF_003018035.1), a genome being accepted as *E. coli* when its average nucleotide identity to the type strain was at least 95%.

### Plasmid replicon screening

Plasmid replicons were identified from assemblies using ABRicate [32] with the PlasmidFinder database [38]. Screening was performed at the nucleotide level, using minimum identity and coverage thresholds consistent with workflow logs. Terminal replicon calls were converted into presence/absence matrices for plotting and summary statistics.

#### Virulence gene detection and gene-matrix construction

Virulence gene screening was performed with ABRicate against an *E. coli* virulence-factor database derived from curated virulence factor resources and literature, using VFDB as a reference framework [39]. The same nucleotide-based thresholding strategy was applied.

To minimize plotting artefacts, gene symbol handling was standardized through case and alias harmonization before creating binary presence/absence matrices and ST-aggregated prevalence summaries. This approach was implemented to prevent false ‘zero’ prevalence results arising from naming inconsistencies, such as *iss* versus *iss2* or *ompT* case variants.

#### Pathotype inference (IPEC/ExPEC and subcategory assignment)

Pathotype inference was conducted using established marker-based logic applied to the virulence-hit tables. For diarrhoeagenic or IPEC categories, canonical regulators, toxins and adhesins were prioritized (e.g. *aggR* for EAEC; *eae* together with *bfpA* and the absence of Shiga-toxin genes for typical EPEC, recognizing that the intimin gene is also present in LEE-positive STEC and EHEC; *stx* for STEC) [40, 41]. For the extraintestinal subpathotypes, no single universally agreed gene set defines each category, so assignment combined the anatomical origin of the specimen with the virulence markers characteristically associated with that subpathotype, following the associations compiled by Sarowska *et al*. [42]. Among the genes screened here, UPEC were recognized by the P-fimbrial adhesins *papC* and *papA*, the iron-acquisition receptors *iutA* and *fyuA* and the toxins *hlyA*, *cnf1* and *sat*; NMEC by the group-2, K1-type capsule marker *kpsM* together with the aerobactin receptor *iutA* and the *sitABCD* iron and manganese system [43]; and sepsis-associated *E. coli* by the serum-survival determinants *iss* and *ompT* with the iron-acquisition receptors *iutA* and *fyuA*. An isolate was assigned to a subpathotype only, where its specimen origin matched the corresponding infection site and the characteristic markers were present, and was designated unassigned otherwise. Isolates were designated as ‘unassigned’ when evidence was insufficient to support a standard label.

### Prophage prediction and prophage-linked virulence screening

Putative prophage regions were predicted using geNomad v1.12.0 (database v1.9) [44] with default settings. Predicted prophage sequences were subsequently screened with ABRicate, using the same thresholds as above, to identify virulence genes located within prophage-associated regions. This approach enabled the distinction, where supported by contig context, between chromosomal or plasmid virulence modules and prophage-linked virulence signals.

### Data aggregation, statistics and figure generation

All outputs, including QC metrics, MLST, phylogroup and serotype assignments, plasmid replicons, virulence hits and geNomad summaries, were aggregated using reproducible shell and Python workflows. Plotting and heatmap generation were performed in Python, utilizing pandas for tabulation and Matplotlib for figure creation. Figures were exported in both vector (SVG) and print-ready (PDF) formats.

## Results

### Genome set and assembly characteristics

Thirty-six *E. coli* draft genomes from Zambia (Sample IDs Zam_UTH_01–Zam_UTH_51, as available) were included in this secondary *in silico* analysis. Specimen metadata and assembly accession information are summarized in Table S1. The isolates originated from a range of clinical specimen types, with stool being the most common (20 out of 36), followed by urine (5 out of 36), pus (4 out of 36), sputum (4 out of 36), high vaginal swab (1 out of 36) and single isolates from blood and CSF (Table S1). The assemblies exhibited characteristics consistent with *E. coli* genomes, including genome sizes ranging from 4.85 to 5.77 Mb (median ~5.38 Mb) and a narrow G+C content range of 50.47–50.96 mol%. Assembly fragmentation was modest (1–17 contigs, median ~9.5 contigs; per-genome assembly metrics are provided in Table S2), which supported subsequent genotyping and gene-content analyses.

### Population structure by Achtman MLST

**Fig. 1.**
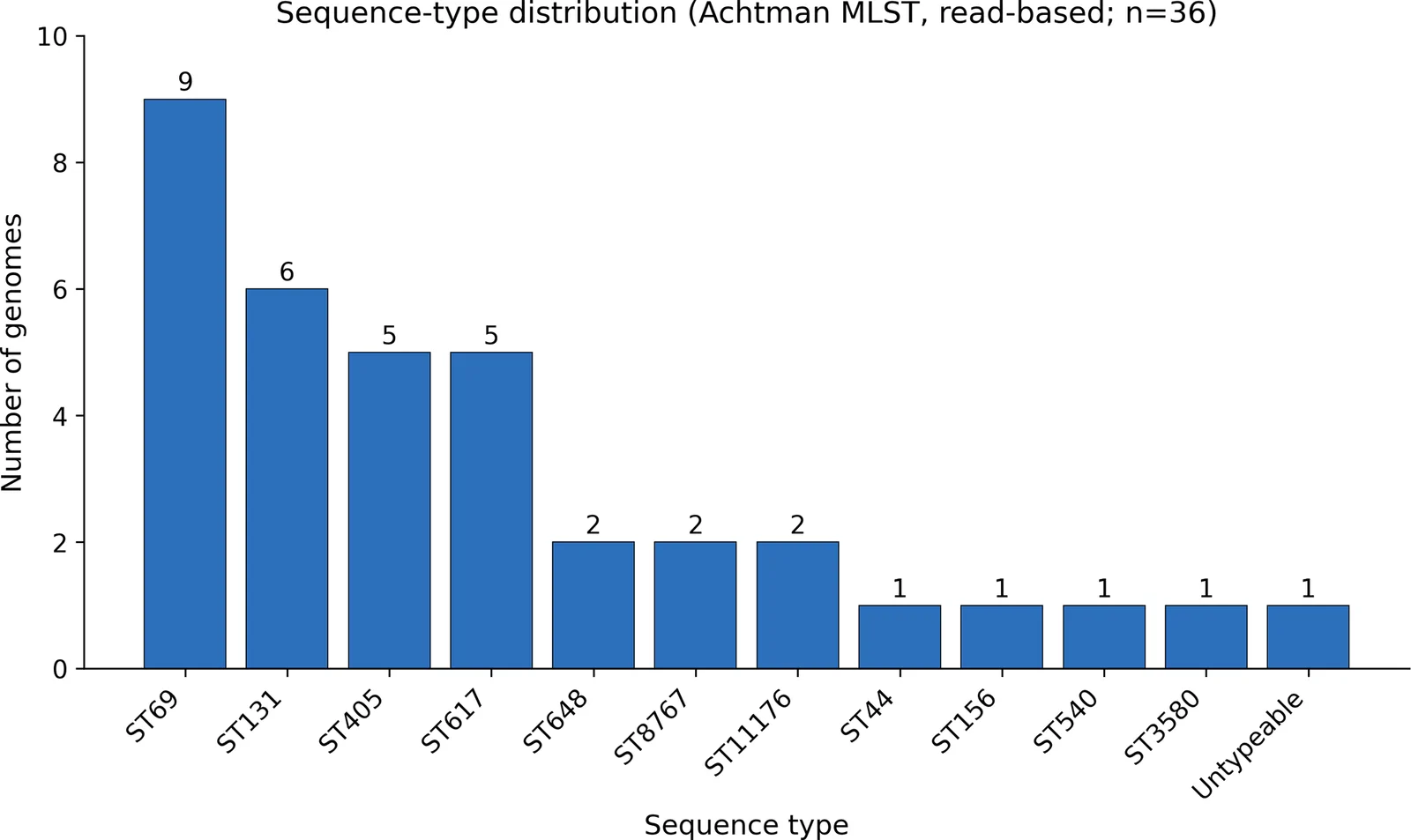
Distribution of STs among 36 *E. coli* draft genome assemblies. The bar chart displays the frequency of multilocus STs assigned from the sequencing reads using the Achtman seven-locus *E. coli* MLST scheme (*adk*, *fumC*, *gyrB*, *icd*, *mdh*, *purA* and *recA*), with assembly-based typing used as a cross-check. Each bar corresponds to a single ST, with the y-axis indicating the number of genomes assigned to that type; values are annotated above the bars. The single genome that could not be assigned a complete seven-locus profile is shown separately as ‘Untypeable’, and single-locus variants are grouped with their parent ST. This distribution characterizes the population structure within the analysed set and facilitates comparison of dominant lineages among isolates.

STs were determined directly from the sequencing reads using the Achtman *E. coli* MLST scheme, with assembly-based typing used as a cross-check. A ST was assigned to 35 of 36 genomes, while a single genome (Zam_UTH_51) remained untypeable owing to a divergent *purA* allele and an unresolved *recA*. Read-based calling recovered loci that were ambiguous in assembly-based typing, predominantly at *recA*, and so resolved isolates that would otherwise have been left unassigned. The most frequent ST was ST69 (nine genomes), followed by ST131 (six genomes), ST405 (five genomes) and ST617 (five genomes). Lower-frequency types comprised ST11176, ST648 and ST8767 (two genomes each) together with the singletons ST156, ST44, ST540 and ST3580 (Fig. 1; Table S3). Two genomes were single-locus variants of their nearest ST. One ST617 genome (Zam_UTH_45) carried a *recA* allele differing by a single nucleotide from its closest curated allele, recA.73, in the Achtman seven-locus *E. coli* scheme (Fig. S1), and one ST69 genome (Zam_UTH_36) carried a *fumC* allele differing from that of ST69; both are reported as single-locus variants and were grouped with their parent ST for tabulation (Fig. 1). As no curated allele identifier was assigned, these are reported as observed sequence differences rather than formally designated new alleles, and the underlying locus sequences are retrievable from the publicly deposited assemblies (Table S1; BioProject PRJDB10450). Per-isolate sequence-type assignments, together with the typing status of any unresolved or variant loci, the assigned phylogroup, the predicted O and H serotype and the average nucleotide identity to the type strain, are provided in Table S3. The sequence-type distribution was concordant with that reported for the same collection by Shawa *et al*. [1], including the shared novel type ST11176, providing independent corroboration of the typing. The globally disseminated pandemic lineage ST131 was the second most frequent type and corresponded to the O25:H4 serotype within phylogroup B2. Overall, these findings indicate a mixed population structure, with enrichment of globally recognized extraintestinal lineages alongside locally diverse backgrounds.

### Phylogroup, serotype and species context

**Fig. 2.**
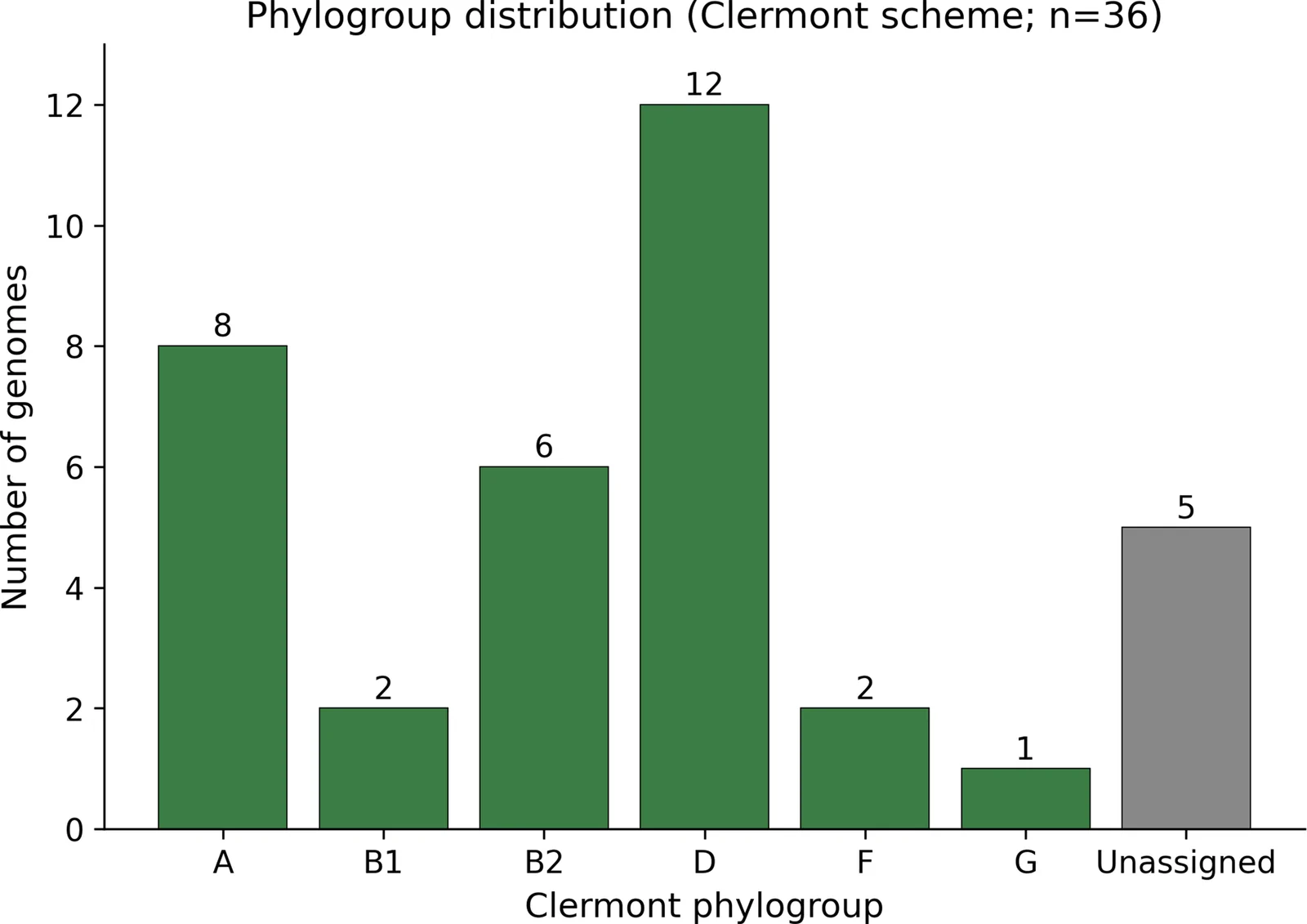
Phylogroup distribution among 36 *E. coli* assemblies. The bar chart shows the distribution of Clermont phylogroups across the 36 genome assemblies. The x-axis lists the phylogroups, and the y-axis indicates the number of genomes assigned to each, with counts annotated above each bar. Phylogroup D was most frequent (*n*=12), followed by A (*n*=8), B2 (*n*=6), B1 (*n*=2), F (*n*=2) and G (*n*=1). Genomes for which one or more Clermont control or typing loci could not be resolved are shown as Unassigned (*n*=5); each such genome nonetheless received a complete ST.

Clermont phylogrouping placed the collection predominantly in extraintestinal-associated phylogroups as depicted in Fig. 2. Phylogroup D was most frequent (12 genomes), followed by phylogroups A (8 genomes), B2 (6 genomes), B1 (2 genomes), F (2 genomes) and G (1 genome). Five genomes (Zam_UTH_03, Zam_UTH_06, Zam_UTH_26, Zam_UTH_34 and Zam_UTH_45) could not be assigned to a phylogroup because one or more Clermont control or typing loci failed to resolve. Each of these genomes nonetheless received a complete ST, so phylogroup-unassigned status reflects the limits of the quadruplex scheme rather than poor data quality. Serotyping revealed distinct patterns across the lineages: the pandemic O25:H4 serotype predominated among phylogroup B2 (ST131) genomes, while an O89:H9 cluster was identified within phylogroup A. Additionally, several phylogroup D (ST69) genomes carried O-antigens spanning the O17, O44, O77 and O106 groups, whereas undetermined O-antigens were classified as such. Species identity was confirmed for all 36 genomes by average nucleotide identity to the *E. coli* type strain (range 96.6 to 98.9%, all above the 95% threshold).

### Virulence gene repertoire and ExPEC signal

Virulence screening across the dataset identified a broad repertoire, with 353 distinct virulence-associated loci detected in total (Table S4a). The number of *E. coli* virulence hits per genome was high but variable (median ~193 genes, range 173–251), indicating substantial accessory virulence content across lineages (Table S4b).

**Fig. 3.**
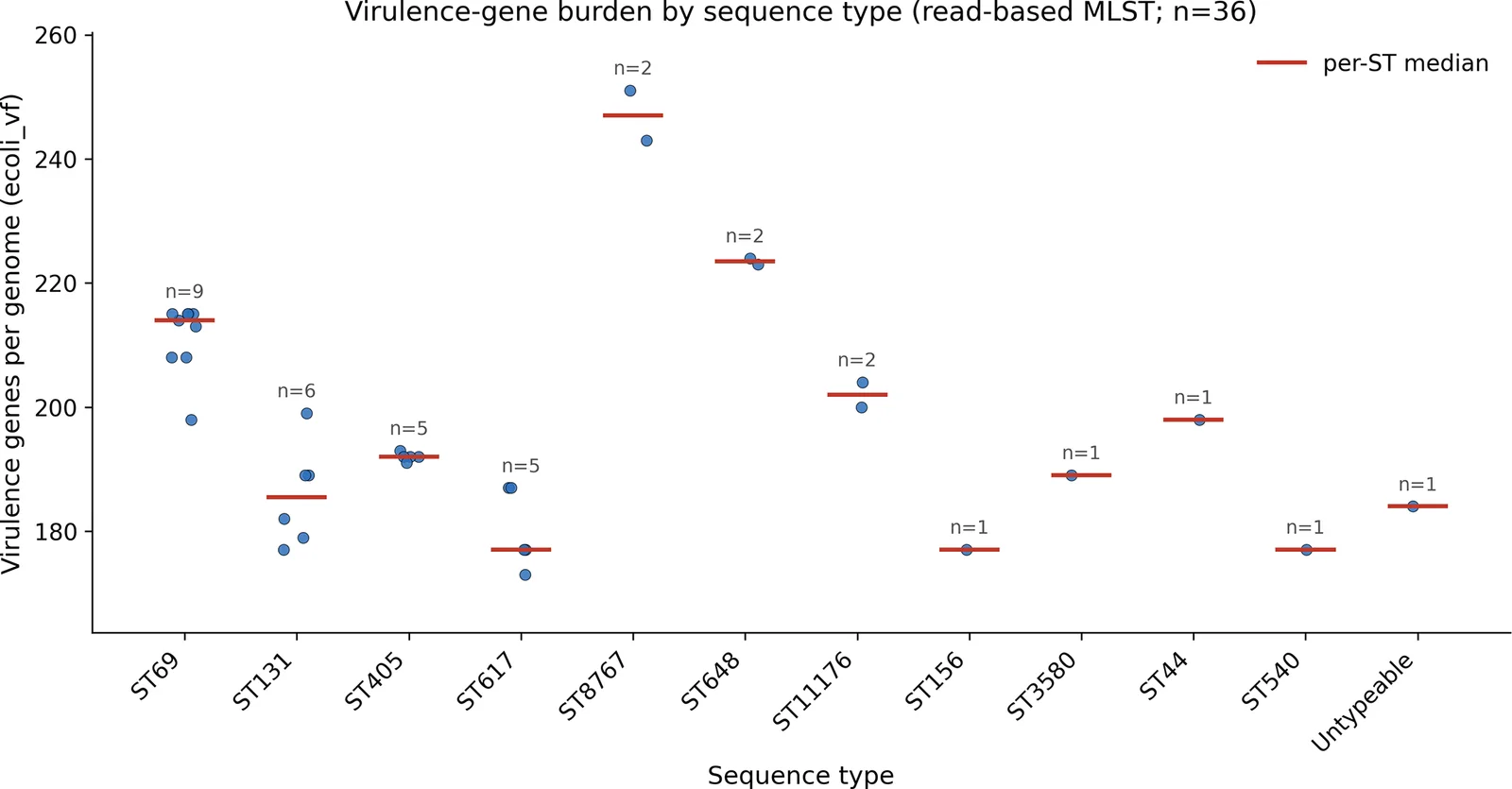
Virulence gene burden by ST among Zambian *E. coli* genomes. Each point represents the number of distinct virulence genes detected in one genome, grouped by Achtman seven-locus MLST ST (scheme: *ecoli_achtman_4*). The horizontal tick within each group marks the per-ST median, and the number of genomes (**n**) contributing to each ST is annotated above each group. Virulence genes are defined as unique gene names identified from the *E. coli* virulence-factor database (*ecoli_vf*) screening results; a gene is considered present in a genome if at least one hit is detected for that gene in the assembly. The single genome that could not be assigned a complete Achtman ST is shown separately as ‘Untypeable’; single-locus variants are grouped with their parent ST.

Restriction of the analysis to a curated panel of clinically interpretable markers showed that the classical IPEC hallmarks *stx1*, *stx2*, *eae*, *bfpA* and *ipaH* were not detected. This finding supports the conclusion that the genome set is not dominated by STEC, EPEC or EIEC signatures. In contrast, multiple ExPEC-associated markers were frequently observed, including *iutA* (27 out of 36), *iss2* (26 out of 36), *fyuA* (24 out of 36), *kpsM* (23 out of 36) and *ompT* (18 out of 36). Lower-frequency detection was noted for *papC* (8 out of 36), *sat* (8 out of 36) and *hlyA*/*cnf1* in a minority of genomes. The distribution of these markers varied by ST, and overall virulence-gene burden also differed between ST groups (Fig. 3).

Application of standard gene-based pathotype classification rules (as detailed in Table S5) indicated that 19 out of 36 genomes met criteria consistent with ExPEC, 2 genomes were classified as EAEC (*aggR*-positive), and 15 genomes lacked sufficient defining markers for assignment to a standard ExPEC or IPEC category (Table S5). When clinical-source-informed subcategories were considered, all urine-derived isolates (5 out of 5) were consistent with a UPEC designation, and single isolates from CSF and blood aligned with NMEC and sepsis-associated ExPEC, respectively (Table S5).

### EAEC-associated loci within an ExPEC-focused dataset

Although the study’s focus is ExPEC virulence profiles, two genomes (Zam_UTH_26 and Zam_UTH_41) carried *aggR* and a concordant EAEC-associated gene set, supporting their classification as EAEC by standard definitions (Table S6). Broader ‘EAEC-associated’ loci were observed beyond these two genomes, most notably *aatA* (12 genomes) and *astA* (10 genomes), but these were largely not accompanied by *aggR*, indicating that the presence of individual EAEC-associated genes in isolation should not be over-interpreted as an indication of EAEC pathotype assignment.

### Plasmid replicon background and linkage to virulence signals

**Fig. 4.**
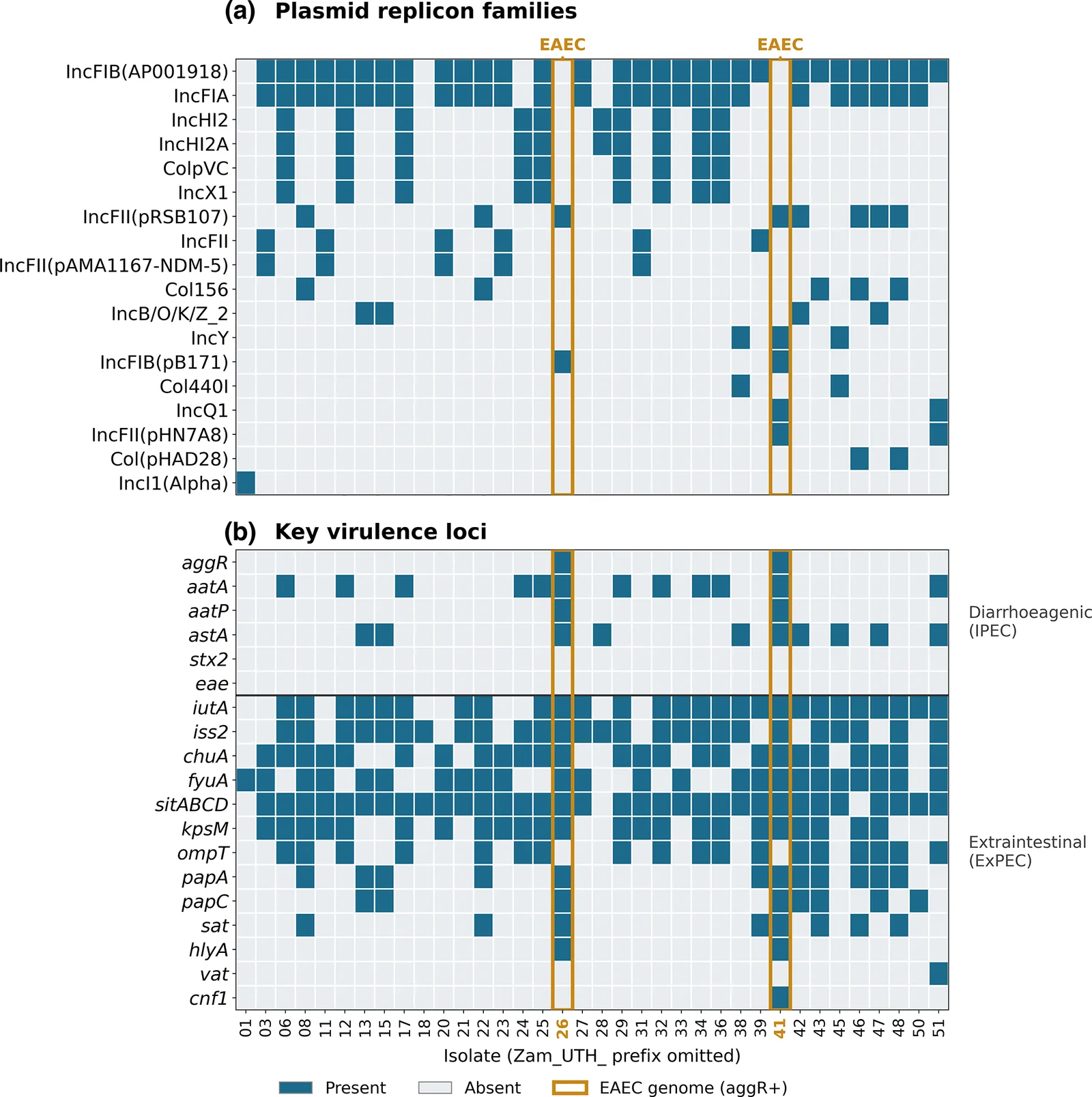
Plasmid replicon families and key virulence loci across the 36 Zambian *E. coli* genomes. For each isolate, filled cells indicate detection of a marker, and unfilled cells indicate its absence. Isolate identifiers on the horizontal axis are abbreviated, omitting the Zam_UTH_ prefix. Panel (a) presents the plasmid replicon families identified *in silico *using ABRicate against the PlasmidFinder database. Replicon families detected in at least two genomes are shown, together with the single IncI1 (Alpha) replicon, ordered by decreasing carriage frequency. Replicon variants detected in only one genome are not displayed. One isolate (Zam_UTH_18) carried no detectable plasmid replicon and is represented by an empty column. Panel (b) presents a set of virulence loci screened with ABRicate against an *E. coli* virulence-factor database (*ecoli_vf*), separated into diarrhoeagenic (IPEC) and extraintestinal (ExPEC) markers. The diarrhoeagenic markers comprise the enteroaggregative-associated determinants *aggR*, *aatA*, *aatP* and *astA*, together with the Shiga-toxin and intimin markers *stx2* and *eae*, both of which were undetected across the collection. The extraintestinal markers comprise *iutA*, *iss2*, *chuA*, *fyuA*, *sitABCD*, *kpsM*, *ompT*, *papA*, *papC*, *sat*, *hlyA*, *vat* and *cnf1*, with *sitABCD* shown as a single row because the 4 operon genes were detected concordantly in every carrier (33 of 36 genomes). The two enteroaggregative *E. coli* (EAEC) genomes, Zam_UTH_26 and Zam_UTH_41, are *aggR*-positive and are outlined and labelled across both panels.

Plasmid replicon screening detected acquired replicons in 35 of 36 genomes, the single exception being Zam_UTH_18, and identified 25 replicon families in total (Fig. 4a; Table S7b). Carriage was dominated by the IncF complex, with IncFIB(AP001918) present in 30 genomes and IncFIA in 27, distributed across all major STs. Among the 30 IncFIB(AP001918) carriers, *sitABCD* was present in 29, *iutA* in 25, *iss2* in 21, *fyuA* in 21 and *kpsM* in 20 (Table S7a).

Several families were largely restricted to single lineages, with IncHI2 and IncHI2A (ten genomes each), IncX1 and ColpVC (nine each) in ST69, an IncFII reference replicon in the five ST405 genomes and Col156 in ST131 (Table S7c).

The remaining families occurred in smaller subsets, and the two *aggR*-positive EAEC genomes (Zam_UTH_26 and Zam_UTH_41) each carried an IncFIB(pB171) replicon detected in no other genome.

### Prophage burden and prophage-linked virulence genes

**Fig. 5.**
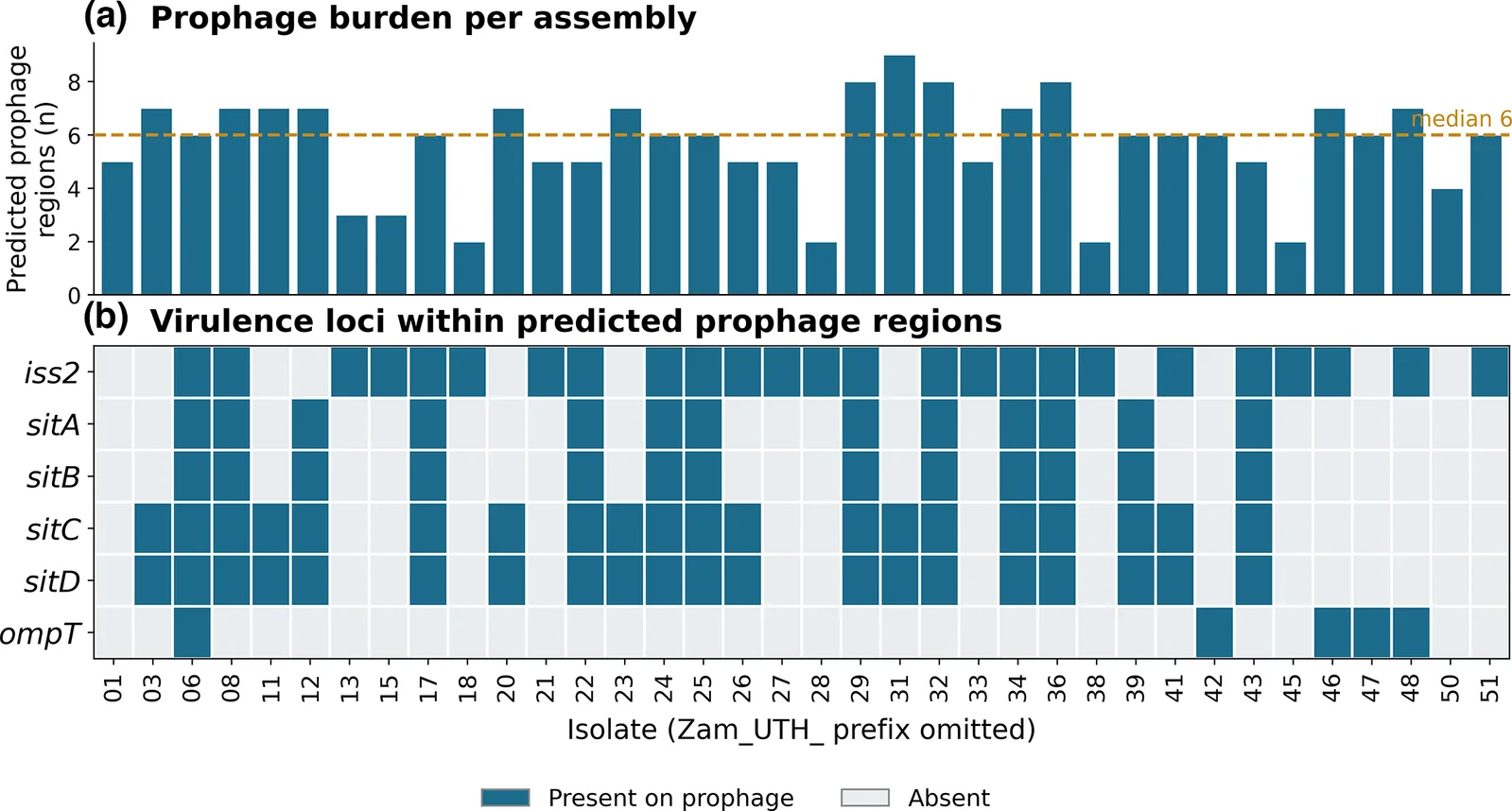
Prophage burden and prophage-associated virulence loci. The top panel (a) presents per-isolate counts of predicted prophage regions (prophage burden) identified from the draft assemblies using geNomad, ranging from two to nine regions per genome (median six). The bottom panel (b) displays a presence/absence heatmap indicating whether each isolate carried at least one predicted prophage region containing the indicated locus, following extraction of prophage sequences and screening for virulence genes (ABRicate against an *E. coli* virulence database). The loci shown comprise the serum-survival determinant *iss2*, the *sitABCD* iron and manganese transport genes and the outer-membrane protease gene *ompT*. The most frequent prophage-borne locus was *iss2* (25 genomes), followed by *sitABCD* (*sitC* and *sitD* in 20 genomes and *sitA* and *sitB* in 13) and *ompT* (5 genomes). All prophage-associated *iss2* copies lay within chromosomal prophage regions bearing viral hallmark genes, whereas *sitABCD* was recovered within chromosomal prophage regions and also outside prophage regions on the chromosome and on IncF plasmids (Table S8). No Shiga-toxin (*stx*) gene was detected within any predicted prophage region. The colour scale indicates presence (1) versus absence (0) per isolate.

The geNomad analysis identified 203 predicted prophage regions across the 36 genomes, with between 2 and 9 regions per genome (median six), so prophage carriage was a general feature of the collection (Fig. 5a). Virulence loci were recovered within at least 1 predicted prophage region in 34 of 36 genomes (Fig. 5b; Table S8a). The most frequent prophage-borne locus was the serum-survival determinant *iss2* (25 genomes), followed by the *sitABCD* iron and manganese transport genes (*sitC* and *sitD* in 20 genomes and *sitA* and *sitB* in 13) and *ompT* (5 genomes), as indicated in Table S8b. No Shiga-toxin (*stx*) gene was detected within any predicted prophage region. Coordinate-level reconciliation of the two recurrent loci against the predicted prophage intervals, stratified by genomic compartment, is summarized in Table S8c. All 25 prophage-associated *iss2* copies lay on chromosomal contigs within predicted prophage regions carrying viral hallmark genes, and no *iss2* copy was recovered from a plasmid contig. *sitABCD* was recovered within predicted prophage regions on chromosomal contigs in 20 genomes and was also present outside prophage regions on the chromosome and on IncF plasmids, with the full compartment split given in Table S8c.

## Discussion

This study set out to characterize the virulence gene content, or virulome, and the mobile accessory genome of ExPEC circulating in Zambia, through a secondary genomic analysis of 36 clinical assemblies generated by Shawa *et al*. [1]. The central observation is that ExPEC-associated virulence genes were both common and broadly distributed, appearing across phylogenetically distinct STs and across a range of specimen types rather than being concentrated in a single pandemic clone. These results indicate that extraintestinal virulence is not confined to a dominant lineage but is instead a modular and distributed property. Within this genetically diverse *E. coli* population, pathogenicity is assembled from shared functional building blocks dispersed via mobile genetic elements.

The value of a virulome-centred analysis in this region follows from a marked imbalance in the existing literature. Globally, genomic attention has concentrated on the pandemic clone ST131, while other extraintestinal lineages have been comparatively neglected [5, 25]. Within Africa, *E. coli* research has been directed largely towards the diarrhoeagenic pathotypes, which reflects the heavy paediatric diarrhoeal burden, and towards AMR surveillance, so that the virulence gene content of extraintestinal isolates has seldom been described [21]. In Zambia, this pattern is evident in the recent focus on diarrhoeagenic and enterotoxigenic isolates from children and on resistance phenotypes [22–24]. By profiling the virulome and the mobilome of an unselected extraintestinal collection, the present analysis begins to address this gap and provides a local baseline for comparison.

Unlike the diarrhoeagenic pathotypes, which are defined by specific toxins and adhesins acting in the gut, ExPEC are defined less by a single marker than by a combination of fitness functions that allow a gut-resident organism to survive and proliferate at normally sterile sites [3, 7]. Foremost among these is iron acquisition, since free iron is scarce in host tissues, and the dataset was dominated by siderophore systems, including aerobactin, signalled by *iutA* (27 out of 36), and yersiniabactin, signalled by *fyuA* (24 out of 36). These systems scavenge host iron and have been shown experimentally to be major determinants of ExPEC virulence [10, 45]. A second function is survival in serum, mediated here by the frequently detected *iss* and *ompT* products, which counter complement-mediated killing [12], and a third is the protective polysaccharide capsule, signalled by *kpsM* (23 out of 36), which resists phagocytosis and complement [46, 47]. Adhesins such as the P fimbriae encoded by *papC* and *papA*, and toxins such as *hlyA*, *cnf1* and *sat*, were present in smaller subsets and contribute to attachment and tissue damage. The implication of this repertoire is substantial. The high prevalence of iron-acquisition, serum-survival and capsule determinants indicates that a large fraction of the circulating Zambian *E. coli* examined here is genetically equipped for extraintestinal survival and that the capacity for invasive disease is a widespread rather than an exceptional feature of this population.

Against this functional background, the population structure was unexpected in an instructive way. Worldwide, a small number of pandemic STs, led by ST131 and including ST69, ST73, ST95, ST127, ST10 and ST38, cause the majority of ExPEC infections, and ST131 has been the single most common type since ~2000 [5]. In the present unselected collection, by contrast, the most frequent type was not ST131 but ST69, with ST131 second. ST69 corresponds to the historic clonal group A, first recognized in 1999 in the USA as a widely disseminated multidrug-resistant cause of UTI, and it belongs to phylogroup D and characteristically carries O-antigens of the O11, O17, O73 and O77 groups [48, 49]. The O17, O44, O77 and O106 antigens recovered among the ST69 genomes here are consistent with that lineage. A predominance of ST69 has clear precedent in some settings, most notably a North American university population in which a single ST69 lineage caused approximately half of trimethoprim-sulfamethoxazole-resistant community UTIs and in which the type has persisted as a community uropathogen for nearly two decades [50]. It nonetheless runs counter to many other collections, since unselected urine series elsewhere are frequently dominated instead by ST73, ST95, ST127 or ST131, as reported for a large Australian collection that was not selected on resistance [51], whereas resistance-selected collections are characteristically dominated by ST131 [25]. The most parsimonious explanation for the present result is therefore methodological as well as biological. The dominance of ST131 is amplified when collections are assembled on an ESBL or other resistance phenotype, because ST131 carries acquired resistance far more often than lineages such as ST69, ST73 and ST95 [52], and removing that selection, as in the unselected collection analysed here, allows a more susceptible but still virulent lineage, such as ST69, to surface as the most frequent type. A local contribution is also plausible, given the previously reported clonal relationship between multidrug-resistant ST69 from poultry and humans in Lusaka, which is consistent with a shared community reservoir [1]. That ST69 also carried the highest median virulence-gene burden in this dataset, and that yersiniabactin contributes to ST69 virulence in experimental UTI and sepsis [45], indicates that its local prominence is unlikely to be incidental and that it warrants surveillance in its own right rather than continued attention to ST131 alone.

The remaining prominent lineages have well-described and contrasting epidemiologies that bear directly on the phylogroup structure considered next. ST131, which represents the second most frequent lineage in this study, is recognized globally as the dominant multidrug-resistant ExPEC clone. Belonging to phylogroup B2 and characterized by the O25:H4 serotype and *fimH*30 allele, ST131 achieved global prominence after 2008 through its strong association with fluoroquinolone resistance and the CTX-M-15 ESBL [25].

ST405, represented by five genomes, is a phylogroup-D high-risk international clone associated with urinary and invasive infection and is a recognized global reservoir of CTX-M-15 and, increasingly, of NDM-type carbapenemases [53–55]. Most phylogroup A genomes identified in this study belonged to ST617, a member of the ST10 clonal complex. Though generally regarded as a commensal host-generalist lacking extraintestinal virulence genes, this complex has spawned globally disseminated, carbapenem-resistant lineages capable of causing severe urinary and BSIs [56, 57]. This distribution highlights a striking trend in pathogenic potential. Traditionally, ExPEC strains are heavily concentrated within phylogroup B2 and, to a lesser extent, phylogroup D, whereas groups A and B1 are regarded as commensal backgrounds with low virulence potential. However, finding a phylogroup D community uropathogen, a pandemic B2 clone, a high-risk phylogroup D resistance reservoir and an emerging clone from a commensal phylogroup A background (ST617) all within this single, modestly sized collection strongly challenges that paradigm. Ultimately, these findings reinforce the central observation: extraintestinal disease in this setting is driven by a multi-clonal architecture of several epidemiologically distinct lineages rather than a single dominant clone [3, 14].

The predominance of phylogroup D (12 of 36), which contains ST69 and ST405, together with the phylogroup-B2 ST131 genomes, is therefore consistent with an extraintestinal-virulence-rich population. Less expected was the substantial phylogroup-A fraction (8 of 36), recovered here from clinical rather than from screening specimens. Two complementary interpretations apply. First, strains of commensal phylogroups are not incapable of causing disease, and phylogroup A and B1 isolates are recovered relatively often from clinical, and particularly healthcare-associated, infections.

Second, and more informative for a virulome analysis, the phylogroup-A isolates in this dataset were not virulence-empty, since the ST617 genomes that constituted the bulk of this group carried the core extraintestinal markers *iutA*, *fyuA* and *iss2* despite belonging to a background ordinarily considered to lack such genes. This pattern is most consistent with the acquisition of extraintestinal fitness functions by an otherwise commensal lineage – a process naturally facilitated by the gut reservoir and subsequent mobile genetic elements [58]. Beyond mere epidemiology, these phylogenetic data demonstrate that functional virulence is not restricted to canonical B2 and D backgrounds; rather, horizontal gene acquisition can transform conventionally commensal lineages into capable extraintestinal pathogens.

The same markers underpin the resolution of the collection into recognized ExPEC subpathotypes, so that the classification follows directly from the gene content described above rather than from clinical labels alone. Applying the criteria detailed in Table S5, 19 of 36 genomes met the predictive definition of an ExPEC pathogen. This outcome was primarily driven by the high prevalence of the aerobactin receptor *iutA* (27 out of 36) and the group 2 capsule marker *kpsM* (23 out of 36), alongside the more variable P-fimbrial adhesin marker *papC* (8 out of 36), all of which are established determinants for assigning extraintestinal status [3]. A further two genomes were assigned to enteroaggregative *E. coli* (EAEC) based on *aggR*, and 15 carried too few defining markers for confident classification.

Combining these gene profiles with specimen origin then yields the site-specific subpathotypes, each best understood not as a separate organism but as the same core machinery deployed at a different anatomical site. All five urine-derived genomes were consistent with UPEC, supported here by the adhesin and iron-acquisition markers *papC*, *papA*, *iutA* and *fyuA*, which together enable attachment to and growth within the urinary tract [59]. The single CSF isolate aligned with NMEC. This subpathotype is characterized by a protective group 2 capsule (classically the K1 type), the aerobactin iron-acquisition system and the *sitABCD* iron/manganese transport system, which collectively enable blood survival and passage across the blood-brain barrier. In the dataset, these three crucial fitness functions were explicitly represented by *kpsM*, *iutA* and *sitABCD*, respectively [43]. The single blood isolate aligned with sepsis-associated ExPEC, in which the serum-survival and iron-acquisition functions marked here by *iss*, *ompT*, *iutA* and *fyuA* are paramount [60]. The recurrent detection of these capsule, iron-acquisition and serum-survival determinants across the dataset is precisely what links the three presentations, since the same genes underlie urinary, meningeal and bloodstream survival.

The discovery of two *aggR*-positive genomes meeting the definition of EAEC within an extraintestinal collection points to a hybrid, heteropathogenic origin rather than laboratory contamination. These strains combine the enteroaggregative pAA plasmid with a classic extraintestinal virulence profile. Such mosaics are increasingly recognized globally, having emerged within the ST131 lineage [61, 62]. Locally, they carry significant regional relevance: identical hybrid configurations, characterized by the co-carriage of the ExPEC markers *iutA*, *fyuA* and *traT*, were recently documented causing BSIs in children from Mozambique [63]. Although the broader enteroaggregative-associated loci *aatA* (12 out of 36) and *astA* (10 out of 36) were detected, they occurred largely in the absence of *aggR*. Consequently, these markers alone do not define the EAEC pathotype; however, their presence is highly consistent with the high baseline of hybrid potential frequently reported among faecal *E. coli* [64].

The mechanism that most economically accounts for this distributed and modular virulence is an active accessory genome dominated by a single plasmid scaffold. IncF-family replicons, principally IncFIB(AP001918) with IncFII, were carried by almost every genome and across all major lineages, and their carriers were strongly enriched for the iron-acquisition, serum-survival and capsule determinants that define extraintestinal fitness. This scaffold corresponds to the conserved virulence plasmidic backbone of extraintestinal and avian-pathogenic *E. coli*, on which *sitABCD*, the aerobactin and salmochelin systems and *iss* are repeatedly co-localized [64]. It is noteworthy that these classes of IncF plasmid backbone are widely recognized as a primary vector for multi-drug resistance cargo, typically facilitating the co-carriage of *blaCTX-M-15* and aminoglycoside, trimethoprim, sulphonamide, macrolide and tetracycline genes to create a unified mobile platform for virulence and resistance [1, 64]. This convergence provides a direct route by which the core extraintestinal virulence functions, together with resistance, can be acquired by diverse and even commensal-associated backgrounds, as observed for the phylogroup-A genomes above.

The accessory genome was also lineage-structured. Alongside the universal IncF scaffold, several replicons were largely confined to single lineages, including IncHI2A and IncX1 in ST69, IncFII variants in ST405 and Col plasmids in ST131. Because isolates of an ST are clonally related and the per-lineage numbers are small, this pattern is most consistent with clonal inheritance rather than independent lineage-specific acquisition, and it may in part reflect local clonal expansion. Aside from ST69, the lineage-restricted IncHI2A and IncX1 backbones were majorly virulence-poor. The limited carriage of virulence genes by these replicon families is echoed by previous studies, which typically attribute a substantial AMR cargo to IncHI2A and IncX1 backbones while noting an absence of classic virulence determinants [65, 66]. Since the present analysis involved some fragmented assemblies, the resulting compartment assignments should be interpreted with caution. They describe the overall architecture of the accessory genome within this collection rather than definitively resolved plasmid configurations, thus warranting future confirmation.

The two mobile compartments were occupied differently. The *sitABCD* system was carried both on IncF plasmids, in keeping with its occurrence on ColV-type virulence plasmids, and within chromosomal prophage regions, whereas *iss2* was recovered almost exclusively within predicted prophage regions and not on plasmids. Prophages were common, at a median of six per genome, and prophage-associated regions are recognized reservoirs of accessory functions even when they carry no classical toxins [26, 28, 67]. Taken together, the plasmid and prophage signals indicate that the virulence repertoire of this population is carried on and shuffled between mobile elements.

These observations, therefore, support a coherent picture of extraintestinal virulence in the Zambian *E. coli* studied. Rather than being restricted to a single pandemic clone, the capacity for invasive disease spans diverse STs and phylogroups, including traditionally commensal backgrounds. Driven by a shared toolkit of iron acquisition, serum survival and capsule functions carried on plasmids and prophages, this pathogenicity manifests clinically as urinary, meningeal or BSIs, as well as in hybrid configurations with enteroaggregative traits. The principal implication is that ExPEC risk in this setting is a population-level and accessory-genome phenomenon that clone-focused surveillance, centred on ST131, is poorly suited to capture. Virulome-aware genomic surveillance, applied alongside the established monitoring of resistance and of diarrhoeagenic pathotypes, would give a more complete account of the extraintestinal threat posed by *E. coli* locally.

Several limitations qualify these interpretations. As a secondary analysis of publicly available draft assemblies, the study is bounded by the original sampling and the available metadata, so genomic findings cannot be linked to clinical syndrome, severity or outcome, and the detection of ExPEC-associated genes should not be equated with disease in any individual isolate. Although the parent isolates derive from the UTH in Lusaka, a national tertiary referral centre that receives specimens from a wide catchment across the country, this remains a single-centre collection, and the population structure and virulence patterns described may not be representative of community settings or of other regions of Zambia, so generalization should be made cautiously. Fragmented draft assemblies constrain assembly-dependent gene and replicon detection, although read-based sequence typing mitigated this limitation for MLST by assigning a type to 35 of 36 genomes. Virulence screening depends on database matches and similarity thresholds and reports gene presence rather than expression. The assignment of genes to specific replicons is least certain in the more fragmented assemblies, and the per-lineage sample sizes are small, so comparisons of virulence content between STs should be regarded as exploratory.

Given the relative scarcity of virulome-level data on extraintestinal *E. coli* in sub-Saharan Africa, these findings underscore the need for larger, prospective and well-curated Zambian datasets. Integrating resistance, virulence and accessory-genome analyses will significantly expand regional ExPEC surveillance, effectively complementing the long-standing focus on diarrhoeagenic pathotypes and resistance phenotypes.

## Supplementary material

10.1099/acmi.0.001165.v3 Supplementary Material 1.

10.1099/acmi.0.001165.v3 Supplementary Material 2.

## Abbreviations

AMR: antimicrobial resistance
BSIs: bloodstream infections
CSF: cerebrospinal fluid
ESBL: extended-spectrum *β*-lactamase
ExPEC: extraintestinal pathogenic *Escherichia coli*
IPEC: intestinal pathogenic *Escherichia coli*
NMEC: neonatal meningitis-associated *Escherichia coli*
QC: quality control
ST: sequence type
UPEC: uropathogenic *Escherichia coli*
UTH: University Teaching Hospital
UTIs: urinary tract infections
