# Supplementary material for: Virulence gene profiles of extraintestinal pathogenic Escherichia coli (ExPEC) from Zambia: a secondary in silico study
Source: Access Microbiol. 2026 Sep 22;8(9):001165.v3. doi: 10.1099/acmi.0.001165.v3 (PMC13597131; doi:10.1099/acmi.0.001165.v3)
Supplement: Supplementary file 1 [file acmi-8-01165-s001.pdf]

SUPPLEMENTARY MATERIALS

A. Nucleotide Sequence Comparison between Isolate *recA* and the closest Reference Allele

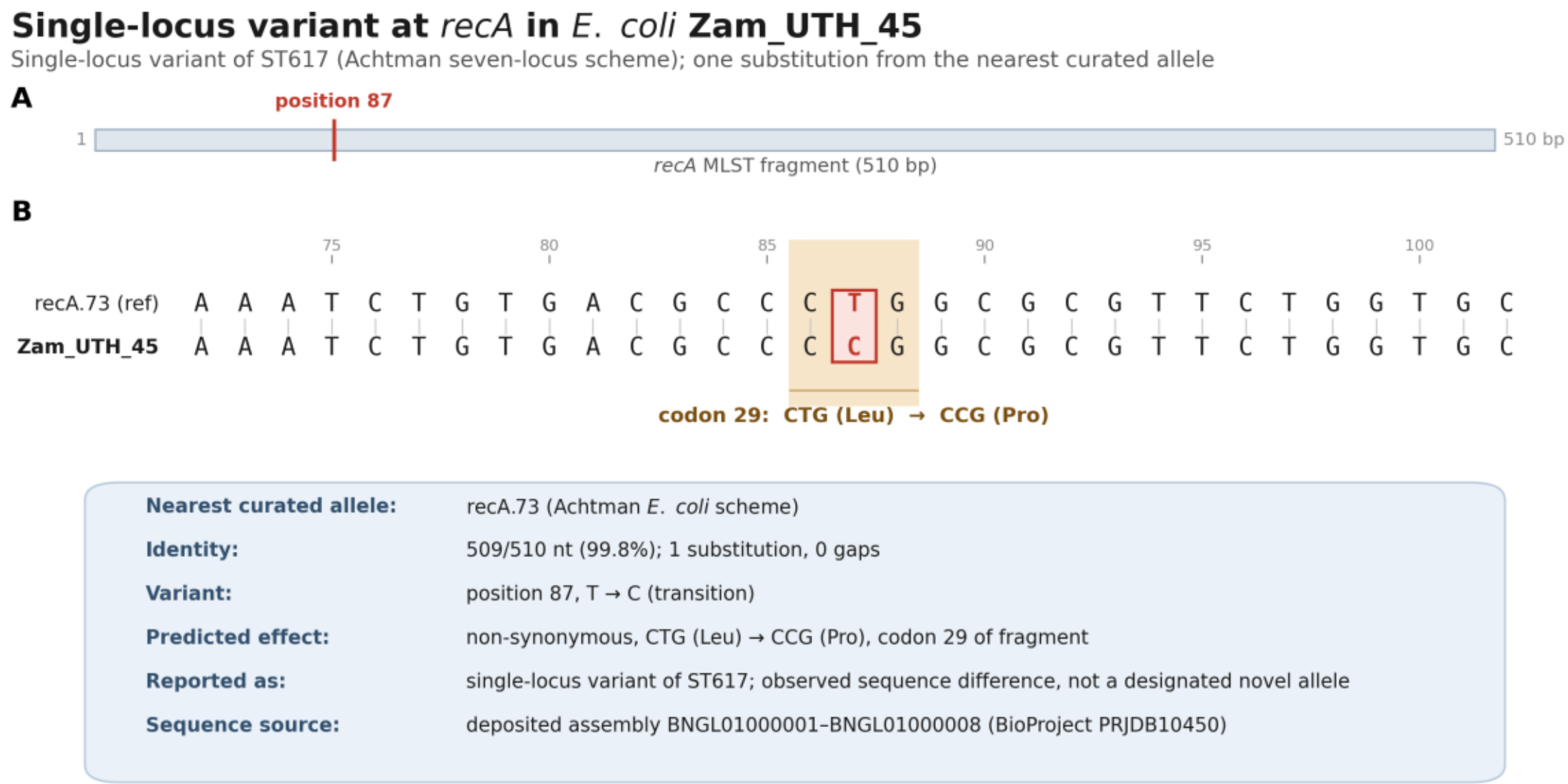

Figure S1. Single-locus variant at recA in Escherichia coli Zam_UTH_45. (A) Location of the single-nucleotide variant within the 510-bp recA fragment of the Achtman seven-locus MLST scheme. **(B)** Pairwise nucleotide alignment between the reference allele (recA.73) and the Zam_UTH_45 sequence (positions 72–102); the single differing position (nucleotide 87) is boxed. The Zam_UTH_45 allele shares 99.8% identity (509/510 bp) with recA.73, differing by a single T-to-C transition at position 87 with no gaps. Within the stop-codon-free reading frame of the typing fragment, this substitution occurs at the second position of codon 29 and is predicted to be non-synonymous (CTG [Leu] to CCG [Pro]). The isolate is classified as a single-locus variant of ST617, with the variation presented as an observed sequence difference rather than a formally designated novel allele. Sequence data are available within the deposited assembly (GenBank accessions BNGL01000001–BNGL01000008; BioProject PRJDB10450).

B. Complete Nucleotide Sequences for the Isolated and Reference *recA* Genes

ISOLATE recA (Zam_UTH_45)

>recA_Zam_UTH_45 nearest=recA.73 pident=99.804 mismatches=1 gaps=0

TGCACGTAAACTGGGCGTCGATATCGACAACCTGCTGTGCTCCCAGCCGGACACT
GGCGAGCAGGCACTGGAAATCTGTGACGCCCCGGCGCGTTCTGGTGCAGTAGAC
GTTATCGTCGTTGACTCCGTGGCGGCACTGACGCCGAAAGCGGAAATCGAAGGCG
AAATCGGCGACTCTCACATGGGCCTTGCGGCACGTATGATGAGCCAGGCGATGCG
TAAGCTGGCGGGTAACCTGAAGCAGTCCAACACGCTGCTGATCTTCATCAACCAG
ATCCGTATGAAAATTGGTGTGATGTTCGGTAACCCGGAAACCACTACCGGTGGTAA
CGCGCTGAAATTCTACGCCTCTGTTCGTCTCGACATCCGTCGTATCGGCGCGGTGA
AAGAGGGCGAAAACGTGGTGGGTAGCGAAACCCGTGTGAAAGTGGTGAAGAAC

AAAATCGCTGCTCCGTTTAAACAGGCTGAATTCCAGATCCTCTACGGCGAAGGTATCAACTTCTATGGCGA

NEAREST REFERENCE ALLELE: recA.73

>recA.73

TGCACGTAAACTGGGCGTCGATATCGACAACCTGCTGTGCTCCCAGCCGGACACTGGCGA

GCAGGCACTGGAAATCTGTGACGCCCTGGCGCGTTCTGGTGCAGTAGACGTTATCGTCGT

TGACTCCGTGGCGGCACTGACGCCGAAAGCGGAAATCGAAGGCGAAATCGGCGACTCTCA

CATGGGCCTTGCGGCACGTATGATGAGCCAGGCGATGCGTAAGCTGGCGGGTAACCTGAA

GCAGTCCAACACGCTGCTGATCTTCATCAACCAGATCCGTATGAAAATTGGTGTGATGTT

CGGTAACCCGGAAACCACTACCGGTGGTAACGCGCTGAAATTCTACGCCTCTGTTCGTCT

CGACATCCGTCGTATCGGCGCGGTGAAAGAGGGCGAAAACGTGGTGGGTAGCGAAACCCG

TGTGAAAGTGGTGAAGAACAAAATCGCTGCTCCGTTTAAACAGGCTGAATTCCAGATCCT

CTACGGCGAAGGTATCAACTTCTATGGCGA

>recA.730

CGCACGTAAACTGGGCGTCGATATCGATAACCTGCTGTGCTCCCAGCCGGACACCGGCGA

GCAGGCACTGGAAATCTGTGACGCCCTGGCGCGTTCTGGCGCAGTAGACGTTATCGTCGT

TGACTCCGTGGCGGCACTGACGCCGAAAGCGGAAATCGAAGGCGAAATCGGCGACTCTCA

CATGGGCCTTGCGGCACGTATGATGAGCCAGGCGATGCGTAAGCTGGCGGGTAACCTGAA

GCAGTCCAACACGCTGCTGATCTTCATCAACCAGATCCGTATGAAAATTGGTGTGATGTT

CGGTAACCCGGAAACCACTACCGGTGGTAACGCGCTGAAATTCTACGCCTCTGTTCGTCT

CGACATCCGTCGTATCGGTGCGGTGAAAGAGGGCGAAAACGTGGTGGGTAGCGA
AACCCG

CGTGAAAGTGGTGAAGAACAAAATCGCTGCGCCGTTTAAACAGGCTGAATTCCAG
ATCCT

CTACGGCGAAGGTATCAACTTCTACGGCGA

>recA.731

CGCACGTAAACTGGGCGTCGATATCGATAACCTGCTGTGCTCCCAGCCGGACACC
GGCGA

GCAGGCACTGGAAATCTGTGACGCCCTGGCGCGTTCTGGCGCAGTAGACGTTATC
GTCGT

TGACTCCGTGGCGGCACTGACGCCGAAAGCGGAAATCGAAGGCGAAATCGGCGA
CTCTCA

CATGGGCCTTGCGGCACGTATGATGAGCCAGGCGATGCGTAAGCTGGCGGGTAAC
CTGAA

GCAGTCCAACACGCTGCTGATCTTCATCAACCAGATCCGTATGAAAATTGGTGTGA
TGTT

CGGTAACCCGGAAACCACTACCGGTGGTAACGCGCTGAAATTCTACGCCTCTGTT
CGTCT

CGACATCCGTCGTATCGGCGCGGTGAAAGAGGGCGAAAACGTGGTGGGTAGCGA
AACTCG

CGTGAAAGTGGTGAAGAACAAAATCGCTGCGCCGTTTAAACAGGCTGAATTCCAG
ATCCT

CTACGGCGAAGGTATCAACTTCTACGGCGA

>recA.732

CGCACGTAAACTGGGCGTCGATATCGACAACCTGCTGTGCTCCCAGCCGGACACT
GGCGA

GCAGGCACTGGAAATCTGTGACGCCCTGGCACGTTCTGGTGCAGTAGACGTTATC
GTCGT

TGACTCCGTGGCGGCACTGACGCCGAAAGCGGAAATCGAAGGTGAAATCGGCGA
CTCTCA

CATGGGCCTTGCGGCACGTATGATGAGCCAGGCGATGCGTAAGCTGGCGGGTAAC
CTGAA

GCAGTCCAACACGCTGCTGATCTTCATCAACCAGATCCGTATGAAAATTGGTGTGA
TGTT

CGGTAACCCGGAAACCACTACCGGTGGTAACGCGCTGAAATTCTACGCCTCTGTT
CGTCT

CGACATCCGTCGTATCGGCGCGGTGAAAGAGGGCGAAAACGTGGTGGGTAGCGA
AACTCG

CGTGAAAGTGGTGAAGAACAAAATCGCTGCGCCGTTTAAACAGGCTGAATTCCAG
ATCCT

CTACGGCGAAGGTATCAACTTCTACGGCGA

>recA.733

CGCACGTAAACTGGGCGTCGATATCGACAACCTGCTGTGTTCCCAGCCGGACACT
GGCGA

GCAGGCACTGGAAATCTGTGACGCCCTGGCACGTTCTGGCGCAGTAGACGTTATC
GTCGT

TGACTCCGTGGCGGCACTGACGCCGAAAGCGGAAATCGAAGGCGAAATCGGCGA
CTCTCA

CATGGGCCTTGCGGCACGTATGATGAGCCAGGCGATGCGTAAGCTGGCGGGTAAC
CTGAA

GCAGTCCAACACGCTGCTGATCTTCATCAACCAGATCCGTATGAAAATTGGTGTGA
TGTT

CGGTAACCCGGAAACCACTACCGGTGGTAACGCGCTGAAATTCTACGCCTCTGTT
CGTCT

CGACATCCGTCGTATCGGCGCGGTGAAAGAGGGTGAAAACGTGGTGGGTAGCGA
AACCCG

CGTGAAAGTTGTGAAGAACAAAATCGCTGCGCCGTTTAAACAGGCTGAATTCCAG
ATCCT

CTACGGCGAAGGTATCAACTTCTACGGCGA

>recA.734

CGCACGTAAACTGGGCGTCGATATCGACAACCTGCTGTGCTCCCAGCCGGACACT
GGCGA

GCAGGCACTGGAAATCTGTGACGCCCTGGCACGTTCTGGTGCAGTAGACGTTATC
GTCGT

TGACTCCGTGGCGGCACTGACGCCGAAAGCGGAAATCGAAGGTGAAATCGGCGA
CTCTCA

CATGGGCCTTGCGGCACGTATGATGAGCCAGGCGATGCGTAAGCTGGCGGGTAAC
CTGAA

GCAGTCCAACACGCTGCTGATCTTCATCAACCAGATCCGTATGAAAATTGGTGTGA
TGTT

CGGTAACCCGGAAACCACTACCGGTGGTAACGCGCTGAAATTCTACGCCTCTGTT
CGTCT

CGACATCCGTCGTATCGGCGCGGTGAAAGAGGGCGAAAACGTGGTGGGTAGCGA
AACCCG

CGTGAAAGTGGTGAAGAACAAAATCGCTGCGCCGTTTAAACAGGTTGAATTCCAG
ATCCT

CTACGGCGAAGGTATCAACTTCTACGGCGA

>recA.735

CGCACGTAAACTGGGCGTCGATATCGACAATCTGCTGTGCTCCCAGCCGGACACC
GGCGA

GCAGGCACTGGAAATCTGTGACGCCCTGGCGCGTTCTGGCGCAGTAGACGTTATC
GTCGT

TGACTCCGTGGCGGCACTGACGCCGAAAGCGGAAATCGAAGGCGAAATCGGCGA
CTCTCA

CATGGGCCTTGCGGCACGTATGATGAGCCAGGCGATGCGTAAGCTGGCGGGTAAC
CTGAA

GCAGTCCAACACGCTGCTGATCTTCATCAACCAGATCCGTATGAAAATTGGTGTGA
TGTT

CGGTAACCCGGAAACCACTACCGGTGGTAACGCGCTGAAATTCTACGCCTCTGTT
CGTCT

CGACATCCGTCGTATCGGCGCGGTGAAAGAGGGCGAAAACGTGGTGGGTAGCGA
AACCCG

CGTGAAAGTGGTGAAGAACAAAATCGCTGCGCCGTTTAAGCAGGCTGAATTCCAG
ATCCT

CTACGGCGAAGGTATCAACTTCTACGGCGA

>recA.736

CGCACGTAAACTGGGCGTCGATATTGACAACCTGCTGTGCTCCCAGCCGGATACC
GGCGA

GCAGGCACTGGAAATCTGTGATGCCCTGGCACGTTCTGGCGCAGTAGACGTTATC
GTCGT

TGACTCCGTGGCGGCACTGACGCCGAAAGCGGAAATCGAAGGCGAAATCGGCGA
CTCTCA

CATGGGCCTTGCGGCACGTATGATGAGCCAGGCGATGCGTAAGCTGGCGGGTAAC
CTGAA

GCAGTCCAACACGCTGCTGATCTTCATCAACCAGATCCGTATGAAAATTGGTGTGA
TGTT

CGGTAATCCGGAAACCACTACCGGTGGTAACGCGCTGAAATTCTACGCCTCTGTTC
GTCT

CGACATCCGTCGTATCGGCGCGGTGAAAGAGGGCGAAAACGTGGTGGGTAGCGA
AACCCG

CGTGAAAGTGGTGAAGAACAAAATCGCTGCGCCGTTTAAACAGGCTGAATTCCAG
ATCCT

CTACGGCGAAGGTATCAACTTCTACGGCGA

>recA.737

CGCACGTAAACTGGGCGTCGATATCGACAACCTGCTGTGCTCCCAGCCGGATACC
GGCGA

GCAGGCACTGGAAATCTGTGACGCCCTGGCACGTTCTGGCGCAGTAGACGTTATC
GTCGT

TGACTCCGTGGCGGCACTGACGCCGAAAGCGGAAATCGAAGGCGAAATCGGCGA
CTCTCA

CATGGGCCTTGCGGCACGTATGATGAGCCAGGCGATGCGTAAGCTGGCGGGTAAC
CTGAA

GCAGTCCAACACGCTGCTGATCTTCATCAATCAGATCCGTATGAAAATTGGTGTGA
TGTT

TGGTAACCCGGAAACCACTACCGGTGGTAACGCGCTGAAATTCTACGCCTCTGTT
CGTCT

CGACATCCGTCGTATCGGCGCGGTGAAAGAGGGCGAAAACGTGGTGGGTAGCGA
AACCCG

CGTGAAAGTGGTGAAGAACAAAATCGCTGCGCCGTTTAAACAGGCTGAATTCCAG
ATCCT

CTACGGCGAAGGTATCAACTTCTACGGCGA

>recA.738

CGCACGTAAACTGGGCGTCGATATCGACAACCTGCTGTGCTCCCAGCCGGATACC
GGCGA

GCAGGCACTGGAAATCTGTGACGCCCTGGCACGTTCTGGCGCAGTAGACGTTATC
GTCGT

TGACTCCGTGGCGGCACTGACGCCGAAAGCGGAAATCGAAGGCGAAATCGGCGA
CTCTCA

CATGGGCCTTGCGGCACGTATGATGAGCCAGGCGATGCGTAAGCTGGCGGGTAAC
CTGAA

GCAGTCCAACACGCTGCTGATCTTCATCAACCAGATCCGTATGAAAATTGGTGTGA
TGTT

CGGTAACCCGGAAACCACTACCGGTGGTAACGCGCTGAAATTCTACGCCTCTGTT
CGTCT

CGACATCCGTCGTATCGGCGCGGTGAAAGAGGGCGAAAACGTGGTGGGTAGCGA
AACCCG

CGTGAAAGTGGTGAAGAACAAAATCGCTGCGCCGTTTAAACAGGCTGAATTCCAG
ATCCT

CTACGGCGAAGGTATCAACTTCTACGGTGA

>recA.739

TGCACGTAAACTGGGCGTCGATATCGACAACCTGCTGTGCTCCCAGCCGGACACT
GGCGA

GCAGGCACTGGAAATCTGTGACGCCCTGGCGCGTTCTGGCGCAGTAGACGTTATC
GTCGT

TGACTCCGTGGCGGCACTGACACCGAAAGCGGAAATCGAGGGCGAAATCGGCGA
CTCTCA

CATGGGCCTTGCGGCACGTATGATGAGCCAGGCGATGCGTAAGCTGGCGGGTAAC
CTGAA

GCAGTCCAACACGCTGCTAATCTTCATCAACCAGATCCGTATGAAAATTGGTGTAA
TGTT

CGGTAACCCGGAAACCACTACCGGTGGTAACGCGCTGAAATTCTACGCCTCTGTT
CGTCT

CGACATCCGTCGTATCGGCGCGGTGAAAGAGGGCGAAAACGTGGTGGGTAGCGA
AACCCG

CGTGAAAGTGGTGAAGAACAAAATCGCTGCGCCGTTTAAACAGGCTGAATTCCAG
ATCCT

CTACGGCGAAGGTATCAACTTCTATGGCGA
